# Symbol-Level Selective Channel Estimation in Packet-Based OFDM Systems

**DOI:** 10.3390/s20051274

**Published:** 2020-02-26

**Authors:** Joo-Young Choi, Cheol Mun, Jong-Gwan Yook

**Affiliations:** 1 Department of Electrical and Electronic Engineering, Yonsei University, Seoul 03722, Korea; 2 Department of Electronic Engineering, Korea National University of Transportation, Chungju 27469, Korea

**Keywords:** correlation coefficient, IEEE 802.11p, orthogonal frequency-division multiplexing (OFDM), selective channel estimation, vehicular environments

## Abstract

Wireless access in vehicular environments to support wireless communication between vehicles has been developed to provide road safety and infotainment services. In vehicular environments where the channel changes rapidly, channel estimation is very important in improving the reliability of wireless communication. Therefore, numerous channel estimation schemes have been proposed; however, none of the schemes proposed so far can perform well over the entire signal-to-noise ratio (SNR) region. In this paper, we propose a novel channel estimation scheme that selectively uses the better scheme between two channel estimation schemes on a symbol-by-symbol basis. The results show that the proposed scheme performs symbol-by-symbol selection of the better channel estimation scheme within a packet, and thus shows excellent performance over the entire SNR region in vehicular environments in terms of the bit error rate and packet error rate.

## 1. Introduction

Cooperative intelligent transportation systems (C-ITS) have been developed to provide various traffic services such as road safety, route planning, and congestion avoidance [1,2,3]. C-ITS is expected to increase traffic efficiency and safety by enabling traffic participants such as vehicles and roadside infrastructures to exchange their information [4]. Wireless communications are perceived as an enabler for high-performance C-ITS applications. Therefore, vehicle-to-vehicle (V2V) and vehicle-to-infrastructure (V2I) communications are key technologies for enabling C-ITSs [5,6]. In particular, wireless access in vehicular environments (WAVE) was designed as a radio communication system for V2V and V2I communications [7,8,9]. In WAVE, the physical (PHY) and medium access control layers are defined by using the IEEE 802.11p and IEEE 802.11p/IEEE 1609.4 standards, respectively [10,11].

C-ITS systems provide safety services for drivers whereby the vehicles periodically transmit a broadcast message containing status information such as their location and speed. In terms of safety services (e.g., cooperative forward collision warning), safety applications can help avoid dangerous road conditions by assisting the driver with the gathered information and sensing problems early enough for the driver to respond [12]. To realize these services, accurate information exchange must be performed, and the reliability of vehicular communications is one of the most important factors. Therefore, robust channel estimation is needed to improve reliability in vehicular environments, and this affects overall system performance.

In high-speed vehicular environments, wireless channels exhibit high Doppler shifts and large delay spreads. Consequently, the channels have both time- and frequency-selective fading owing to motion and multipath components [13,14,15]. The channel conditions vary rapidly and affect the transmitted packet; thus, the channel coefficient can change significantly from the start to the end of the packet. To adapt to these rapid channel variations, IEEE 802.11p/WAVE employs four pilot subcarriers among 48 data subcarriers in a packet. However, these pilot subcarriers are not sufficient for estimating channel variations in the frequency domain owing to large delay spreads; consequently, performance degradation occurs in communication systems.

Therefore, the accuracy of channel estimation has a significant effect on system performance, and numerous channel estimation schemes complying with the IEEE 802.11p standard have been proposed to adapt to fast-varying channels [16,17,18,19,20,21]. A good survey and review of other schemes can be found in [21]. The spectral temporal averaging (STA) [16] scheme can demonstrate better error performance at low signal-to-noise ratios (SNRs), but an error floor occurs at high SNRs. The time-domain reliable test frequency domain interpolation (TRFI) scheme [17] improves the channel estimation performance using the time-frequency correlation properties, but it cannot achieve satisfactory performance at low SNRs. In [18], the decision-directed estimation with time-domain truncation (DD-TT) channel estimation scheme was proposed; however, performance degradation occurs at high SNRs. In [19], channel estimation using frequency self-organization was proposed to improve error performance. This method needs a decoder and encoder chain equal to the number of symbols in the packet to obtain the channel estimate, resulting in high computational complexity and delay. In [20], the packet-level selective (PLS) scheme, which selects the better channel estimation scheme on a packet-by-packet basis, was proposed by our researchers to achieve outstanding performance over the entire SNR region. However, as this technique uses long preambles only once at the start of the packet to determine the better channel estimation technique, the PLS scheme has difficulty in accurately tracking channel variations that occur along the packet over time. Thus, to improve the performance of the channel estimation, it is necessary to track the channel variations within a packet caused by high Doppler shifts in vehicular communications.

Through a comprehensive analysis of existing techniques, our main motivation is to design a novel channel estimation scheme that can accurately track channel variations from the beginning to the end of a packet in a vehicular environment, where high mobility produces rapid channel variations within a packet. Therefore, in this paper, we propose a symbol-level selective (SLS) channel estimation scheme that selectively uses two channel estimation schemes—TRFI and DD-TT, which demonstrate good performance in different disjointed SNR regions—on a symbol-by-symbol basis in a packet. As TRFI and DD-TT demonstrate superior performance in high- and low-SNR regions, respectively, if these techniques can be adaptively selected for each symbol in a packet, then the error performance can be better than that of the conventional technique.

The channel estimation proposed in this paper is not a simple combination of DD-TT and TRFI, but the creation of a new channel estimation scheme selecting the better one between DD-TT and TRFI on the symbol-by-symbol basis without significantly increasing the computational complexity. A simple combining method may select the better one by comparing the error performance of the two techniques after applying both techniques at the same time on the received packet, which leads to significant computational complexity. As a result, the simply combining method introduces a delay in choosing a better technique, which degrades the system performance. Overall, our contributions in this paper are as follows:

- We present a new criterion that selects a better channel estimation scheme between two channel estimation schemes (DD-TT and TRFI) on a symbol-by-symbol basis without significantly increasing the computational complexity.
- We perform a complexity analysis of the proposed scheme to demonstrate its implementation feasibility and prove that additional computational complexity is not substantial while selecting a better technique.
- We analyze the trade-off between performance and complexity according to the algorithm’s selection period within a packet.
- By providing simulation results in terms of the bit error rate (BER) and packet error rate (PER), we demonstrate that the better channel estimation scheme can be selected on a symbol-by-symbol basis, thereby obtaining a performance gain in vehicular environments.

The rest of the paper is organized as follows. In Section 2, IEEE 802.11p and the channel models adopted in the simulations are briefly introduced. Section 3 is a concise overview of three practical channel estimation techniques: DD-TT, TRFI, and PLS. In Section 4, we describe the algorithm for the proposed SLS scheme. Section 5 performs a computational complexity analysis of the proposed algorithm. Section 6 analyzes the simulation results for a performance comparison. Finally, in Section 7, we provide our conclusions.

## 2. System Model

### 2.1. IEEE 802.11p

IEEE 802.11p is based on orthogonal frequency-division multiplexing (OFDM). OFDM improves the spectral efficiency and mitigates the severity of multipath fading by modulating the encoded data to *N* parallel orthogonal subcarriers for transmission. The modulation and demodulation of OFDM are performed through inverse fast Fourier transform and fast Fourier transform (FFT), respectively. OFDM inserts a guard interval of length *G*, called a cyclic prefix (CP), in the time domain, which avoids intersymbol interference and intercarrier interference between OFDM symbols. An OFDM symbol occupies a bandwidth *B*, and the sampling time is $T_{s}=1/B$ at the receiver. Thus, the duration of an OFDM symbol is defined as $T_{OFDM}=\left(N_{tot}+G\right)T_{s}$. The specific system parameters are listed in Table 1. The transmission is performed by framing *M* OFDM symbols, which can vary depending on the desired payload size. The transmitter utilizes a convolutional encoder, and the receivers adopt the Viterbi decoder.

An IEEE 802.11p packet consists of a preamble, signal field, and data field. In particular, two long training symbols in the preamble, which are located at the beginning of the packet, are used for channel estimation. The data field is composed of a number of data symbols, and each OFDM symbol consists of 64 subcarriers with indexes ranging from −32 to 31. Of the 64 subcarriers, those with indexes −21, −7, 7, and 21 are allocated as pilot symbols, and those with indexes from −26 to 26 are allocated to 48 data subcarriers, excluding those with indexes −21, −7, 0, 7, and 21. The remaining 12 indexes are set to null subcarriers.

**Figure 1:**
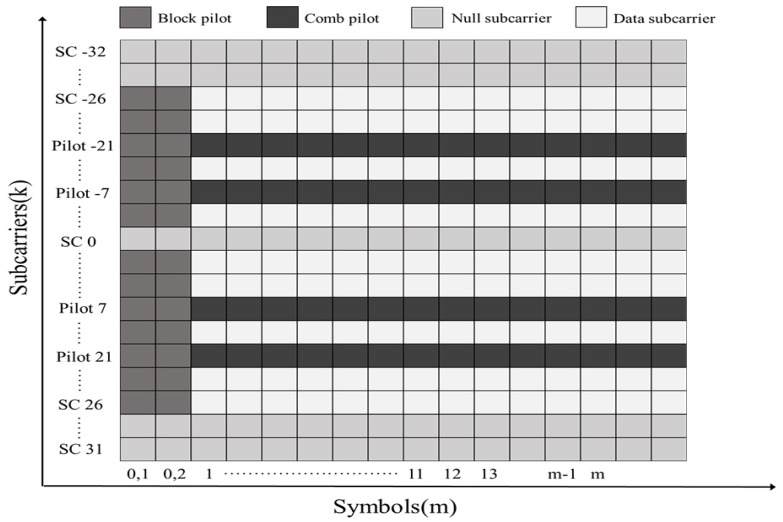
Pilot arrangement of IEEE 802.11p.

The 802.11p standard uses a block-comb-type pilot pattern for channel estimation, as shown in Figure 1. The two symbols at the beginning of the IEEE 802.11p packet are the block pilot, and each OFDM symbol then uses the pilot as the comb type. However, the pilot subcarriers specified in the standard are not sufficient to track frequency-selective fading channels, resulting in a performance degradation of the system.

### 2.2. Channel Model

In realistic vehicular environments, the performance of the communication system relies on the condition of each communication link. That is, reliable vehicular communication is dependent on the channel conditions [14]. The challenging properties of wireless channels have a significant impact on the performance that can be achieved in communication systems; thus, analyzing the characteristics of a wireless channel is key to improving the system performance. For this reason, many studies and measurements of vehicular wireless channels have been performed [13,14,15,22,23,24,25,26,27].

In this study, the vehicular channel models presented in [23,24] are implemented in a simulation. The channel models for [23,24] are designed for standardization as channel models for V2V and V2I communications in high-speed vehicles. These channel models are classified into six scenarios according to V2V and V2I environments. These six channel models are schematized as a tapped delay line (TDL), including the number of taps, tap power, and delay value. The TDL model is commonly employed to model multipath channels, and it is easy to implement in a simulator. Therefore, TDL models are widely used for evaluating system performance.

Of the six models, V2V Expressway Oncoming and V2I Urban Canyon in Table 2 are considered because of their importance in real situations. The two models represent a typical vehicular environment. A specific summary of channel models such as TDL and the Doppler frequency for each channel tap is provided in [24].

## 3. Related Work

In the receiver, assuming perfect timing and frequency synchronization, the received symbol at subcarrier *k* in symbol *m* after removing the CP and performing an FFT can be expressed as follows:

(1) $$\begin{array}{c} Y\left(m,k\right)=H_{p}\left(m,k\right)X\left(m,k\right)+Z\left(m,k\right), \end{array}$$

where $H_{p}\left(m,k\right)$ is the perfect channel response (CR), and Z(m,k) is additive white Gaussian noise with zero mean and variance $\sigma_{z}^{2}$, i.e., $N(0,\sigma_{z}^{2})$.

To track channel variations in a time-varying channel, the DD channel estimation scheme is widely used and continuously updates the channel estimates [28]. First, the *m*-th frequency-domain received symbol Y(m,k) is compensated by the (m−1)-th channel estimate H(m−1,k) as follows:

(2) $$\hat{T}\left(m,k\right)=\frac{Y(m,k)}{H(m-1,k)},m=1,\cdots ,M.$$

In particular, when m=1, H(0,k) is defined as the average channel estimate obtained by dividing the first two received symbols $Y_{0}\left(1,k\right)$ and $Y_{0}\left(2,k\right)$ by the known long training symbol $X_{0}\left(k\right)$, i.e., $H\left(0,k\right)=\frac{Y_{0}\left(1,k\right)+Y_{0}\left(2,k\right)}{2X_{0}\left(k\right)}$.

Thus, $\hat{X}\left(m,k\right)$ is obtained by demapping $\hat{T}\left(m,k\right)$ in accordance with the modulation scheme, and $\hat{X}\left(m,k\right)$ is again employed to obtain an initial channel estimate $\hat{H}\left(m,k\right)$.

(3) $$\begin{array}{cc} \hat{H}\left(m,k\right) & =\frac{Y(m,k)}{\hat{X}\left(m,k\right)}\\ \; & =\frac{H_{p}\left(m,k\right)X\left(m,k\right)}{\hat{X}\left(m,k\right)}+\frac{Z(m,k)}{\hat{X}\left(m,k\right)}. \end{array}$$

$\hat{H}\left(m,k\right)$ obtained from Equation (3) still includes both the demapping error and noise component. Therefore, various channel estimation schemes have been proposed to remove the error components of $\hat{H}\left(m,k\right)$ effectively.

In this section, we outline three channel estimation schemes of TRFI, DD-TT, and PLS, which are related to the proposed scheme in this paper.

### 3.1. TRFI Channel Estimation Scheme

TRFI employs the frequency-time correlation characteristics of adjacent subcarriers and symbols. First, the (m−1)-th received symbol Y(m−1,k) is divided by the channel estimate obtained from Equation (3).

(4) $${\hat{T}}^{\prime }\left(m-1,k\right)=\frac{Y(m-1,k)}{\hat{H}\left(m,k\right)}.$$

Subsequently, the (m−1)-th channel estimate H(m−1,k) is used to divide Y(m−1,k).

(5) $${\hat{T}}^{\prime \prime }\left(m-1,k\right)=\frac{Y(m-1,k)}{H(m-1,k)}.$$

${\hat{T}}^{\prime }\left(m-1,k\right)$ and ${\hat{T}}^{\prime \prime }\left(m-1,k\right)$ are demapped into ${\hat{X}}^{\prime }\left(m-1,k\right)$ and ${\hat{X}}^{\prime \prime }\left(m-1,k\right)$ in accordance with the modulation scheme, and the demapped symbols are subjected to a time reliability test. If the demapped symbols are matched to the same symbol, then the initial channel estimate $\hat{H}\left(m,k\right)$ is determined as the final channel estimate H(m,k). Meanwhile, the final channel estimate H(m,k) that fails to pass the test is obtained through frequency interpolation using the channel estimates that have passed the test.

The performance of TRFI highly depends on the accuracy of the initial channel estimate. As the SNR increases, the smaller demapping error improves the accuracy of the initial channel estimate. This enhances the accuracy of the channel estimates obtained by the time reliability test and frequency interpolation; thus, TRFI provides high performance. By contrast, as the SNR decreases, the demapping error caused by a considerable noise component lowers the accuracy of the initial channel estimate, which leads to a performance degradation.

### 3.2. DD-TT Channel Estimation Scheme

The DD-TT scheme effectively removes the noise component in the initial channel estimate $\hat{H}\left(m,k\right)$ using time-domain truncation, thereby improving the channel estimation performance.

First, $\hat{H}\left(m,k\right)$ is represented as a column vector.

(6) $$\begin{array}{c} {\hat{H}}_{m}=[\hat{H}\left(m,-26\right),\hat{H}\left(m,-25\right),\hat{H}\left(m,k\right)\\ \cdots \;\hat{H}{\left(m,26\right)]}^{T},\;k\in K, \end{array}$$

where K defines the index set of data and comb pilot subcarriers.

Subsequently, the frequency-domain channel vector is converted into a time-domain channel vector as follows:

(7) $${\hat{h}}_{m}=\frac{1}{K}W^{H}{\hat{H}}_{m},$$

where W is a matrix obtained by converting the number of columns of the FFT matrix of size K×K into *L*, i.e., W is a K×L matrix. W is expressed such that

(8) $$W=\sqrt{K}F_{K}\left(:,1:L\right),$$

where *L* represents the channel order. In general, the delay spread in vehicular environments is smaller than the guard interval *G*. Hence, the channel impulse response can be limited to a specific number *L*. In [18], *L* is set to 8 considering the V2V channel model of [23].

The time-domain channel vector is the value of the channel in which the noise components after the *L*-th tap are removed and indicated, as follows:

(9) $$\hat{h}\left(m,n\right)=\left\{\begin{array}{cc} h(m,n)+z(m,n), & n=1,2,3,\cdots ,L\\ 0, & \mathrm{otherwise}, \end{array}\right.$$

where *n* denotes the time sample, and h(m,n) and z(m,n) represent the channel and noise components in the time domain, respectively.

The final channel estimate vector in the frequency domain can be obtained via time-domain truncation such that

(10) $$H_{m}=W{\hat{h}}_{m}\left(1:L\right).$$

The DD-TT improves performance by eliminating unnecessary noise components after the *L*-th tap in the time domain. At low SNRs, the effect on noise is larger, so the performance improvement caused by the noise component removal is outstanding. However, as the SNR increases, the effect of noise is reduced. This leads to the removal of necessary channel information and thus generates an error floor.

### 3.3. PLS Channel Estimation Scheme

The PLS scheme selectively uses the better channel estimation scheme of two candidate schemes with good performance in their corresponding SNR regions on a packet-by-packet basis [20]. First, the long training symbols are used to obtain the channel estimate H(0,k) defined in Section 3. $H_{i}\left(0,k\right)$ is then obtained by using a candidate channel estimation technique *i* in H(0,k). The modified mean squared error (MSE) for the candidate channel estimation scheme is defined as follows:

(11) $${\tilde{\delta }}_{i}=\frac{1}{N}\sum_{k=-26}^{26}{\left|H\left(0,k\right)-H_{i}\left(0,k\right)\right|}^{2},$$

where *N* is the total number of data and pilot subcarriers in each OFDM symbol. The selection criterion is then defined as follows:

(12) $$\zeta_{i}=\underset{i\in C}{arg min}\left|\frac{1}{\bar{\gamma }}-{\tilde{\delta }}_{i}\right|.$$

Here, C denotes a set of two candidate schemes, and $\bar{\gamma }$ represents the average SNR. By finding *i* that minimizes $\left|1/\bar{\gamma }-{\tilde{\delta }}_{i}\right|$ in C, the PLS scheme can choose a better technique. The selected channel estimation scheme using the preamble is employed to estimate the channel of all data symbols in the packet.

Assuming that $H_{i}\left(0,k\right)$ is similar to a perfect CR, the modified MSEs are defined as $1/\bar{\gamma }$. Therefore, the better the channel estimation scheme between the candidate schemes, the closer the channel estimate is to the perfect CR, and ${\tilde{\delta }}_{i}$ of the better scheme is approximated to $1/\bar{\gamma }$.

The PLS scheme achieves a performance gain by selectively using two channel estimation schemes with superior performance in different SNR regions. However, the selected channel estimation scheme at the beginning of a packet has difficulty in precisely estimating the channel variations within the packet over time in vehicular environments. In addition, because the method of selecting a better channel estimation scheme in the PLS scheme is based on the long preamble in front of the packet, it cannot be extended to a scheme for changing the channel estimation scheme in the middle of the packet.

## 4. Selective Channel Estimation Based on Correlation Coefficient

In this section, we propose a criterion for selecting the better channel estimation scheme between the two channel estimation schemes on a symbol-by-symbol basis. TRFI and DD-TT show good performance at high and low SNRs, respectively. Therefore, if the better channel estimation scheme can be selected for each symbol rather than an entire packet, then the channel variations from the beginning to the end of the packet can be accurately tracked. Thus, the performance can be better than that of conventional techniques.

**Figure 2:**
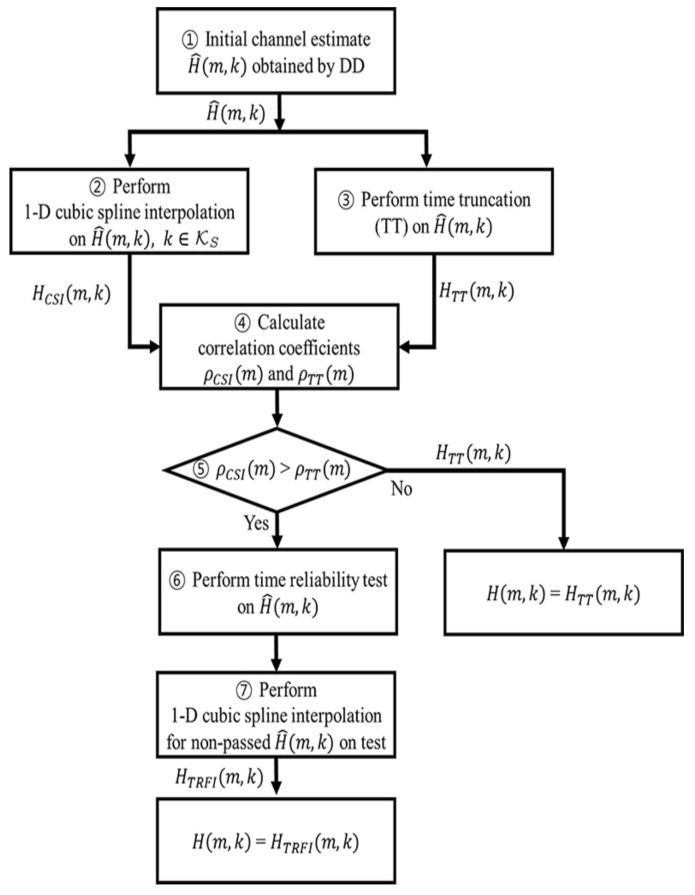
Flowchart of the proposed SLS scheme.

Figure 2 shows a flowchart of the proposed SLS scheme. First, in Step ①, the initial channel estimate of the *m*-th OFDM symbol in a packet is obtained as $\hat{H}\left(m,k\right)$ by computing Equations (2) and (3).

Subsequently, in Steps ② and ③, cubic spline interpolation (CSI) and TT are performed on initial channel estimate $\hat{H}\left(m,k\right)$ to obtain $H_{CSI}\left(m,k\right)$ and $H_{TT}\left(m,k\right)$, respectively. $H_{CSI}\left(m,k\right)$ obtained through the CSI on the initial channel estimates is used to select the TRFI. To obtain $H_{CSI}\left(m,k\right)$, first, we define the uniformly spaced subcarriers indexed as $K_{S}≜\left\{\pm 2,\;\pm 5,\;\pm 7,\;\pm 10,\;\pm 14,\;\pm 18,\;\pm 21,\;\pm 24\right\}$. $K_{S}$ is designated as the pilot subcarrier indexes and data subcarrier indexes spaced at regular intervals from the pilot subcarriers in consideration of correlation with the initial channel estimate. For $\forall k\in K_{S}$, the channel estimates of subcarrier *k* are obtained by CSI using $\hat{H}\left(m,k\right)$, $\forall k\in K\backslash K_{S}$. Meanwhile, $H_{TT}\left(m,k\right)$ is obtained by performing Equations (6), (7), and (10) on initial channel estimate $\hat{H}\left(m,k\right)$.

In Step ④, we calculate the correlation coefficient between $\hat{H}\left(m,k\right)$ and $H_{j}\left(m,k\right)$ for indexes $K_{S}$, where *j* represents CSI or TT. The correlation coefficient $\rho_{j}\left(m\right)$ is defined as follows:

(13) $$\rho_{j}\left(m\right)=\frac{1}{R-1}\left|\sum_{k\in K_{S}}\left(\frac{\bar{\hat{H}\left(m,k\right)-\mu_{\hat{H}}}}{\sigma_{\hat{H}}}\right)\left(\frac{H_{j}\left(m,k\right)-\mu_{H_{j}}}{\sigma_{H_{j}}}\right)\right|,$$

where $\rho_{j}\left(m\right)$ denotes the correlation coefficient between $\hat{H}\left(m,k\right)$ and $H_{j}\left(m,k\right)$ at the *m*-th OFDM symbol, and $\bar{\left(\cdot \right)}$ represents a complex conjugate. $\mu_{\hat{H}}$ and $\sigma_{\hat{H}}$ are the mean and standard deviation of $\hat{H}\left(m,k\right)$, respectively, and $\mu_{H_{j}}$ and $\sigma_{H_{j}}$ are the mean and standard deviation of $H_{j}\left(m,k\right)$, respectively. *R* is the number of indexes in $K_{S}$.

Finally, in Step ⑤, if the correlation coefficient of CSI, $\rho_{CSI}$, is higher than that of TT, $\rho_{TT}$, then the final channel estimate H(m,k) of the *m*-th OFDM symbol is determined as the channel estimate $H_{TRFI}\left(m,k\right)$ obtained by performing the TRFI (Steps ⑥ and ⑦). Otherwise, the final channel estimate H(m,k) is determined as the channel estimate $H_{TT}\left(m,k\right)$. This process is performed sequentially on *M* OFDM symbols constituting one packet.

The correlation coefficient can determine the degree of correlation between two variables. $H_{CSI}\left(m,k\right)$ and $H_{TT}\left(m,k\right)$ are based on the initial channel estimate; thus, by analyzing the correlation coefficients between the initial channel estimate $\hat{H}\left(m,k\right)$ and two channel estimates, i.e., $H_{CSI}\left(m,k\right)$ and $H_{TT}\left(m,k\right)$, the better channel estimation technique can be selected.

In low-SNR regions, $\rho_{TT}\left(m\right)$ becomes larger than $\rho_{CSI}\left(m\right)$ for the following reason:

(14) $$\begin{array}{cc} \; & \mathrm{In}\;\mathrm{the}\;\mathrm{limit}\;\gamma \to 0\\ \; & \Rightarrow H_{CSI}\left(m,k\right)≁\hat{H}\left(m,k\right)\;∵\mathrm{large}\;\mathrm{demapping}\;\mathrm{error}\\ \; & \Rightarrow H_{TT}\left(m,k\right)∼\hat{H}\left(m,k\right)\\ \; & ∵h_{TT}\left(m,n\right)=\hat{h}\left(m,n\right),\;n=1,2,3,\cdots ,L\\ \; & ∴\rho_{CSI}\left(m\right)<\rho_{TT}\left(m\right), \end{array}$$

where γ represents the SNR, and ∼ denotes “similar in distribution.” In low-SNR regions, a large demapping error owing to a considerable noise component lowers the accuracy in the initial channel estimate $\hat{H}\left(m,k\right)$. Moreover, frequency interpolation on incorrect channel estimates causes the propagation of uncertainty in the channel estimate $H_{CSI}\left(m,k\right)$, resulting in a low correlation coefficient between $H_{CSI}\left(m,k\right)$ and $\hat{H}\left(m,k\right)$. By contrast, as shown in Equation (9), $H_{TT}\left(m,k\right)$ obtained by DD-TT includes most channel components of the initial channel estimate $\hat{H}\left(m,k\right)$ within the *L*-th tap of $H_{TT}\left(m,k\right)$. This results in a higher correlation coefficient with the initial channel estimate than $H_{CSI}\left(m,k\right)$.

Conversely, in high-SNR regions, $\rho_{CSI}\left(m\right)$ is larger than $\rho_{TT}\left(m\right)$ for the following reason:

(15) $$\begin{array}{cc} \; & \mathrm{In}\;\mathrm{the}\;\mathrm{limit}\;\gamma \to \infty \\ \; & \Rightarrow H_{CSI}\left(m,k\right)∼\hat{H}\left(m,k\right)\;∵\mathrm{decreasing}\;\mathrm{demapping}\;\mathrm{error}\\ \; & \Rightarrow \rho_{CSI}\left(m\right)\approx 1\\ \; & \Rightarrow \rho_{TT}\left(m\right)<1\;∵h_{TT}\left(m,n\right)=\hat{h}\left(m,n\right)=0,\;\mathrm{after}L-\mathrm{tap}\\ \; & ∴\rho_{CSI}\left(m\right)>\rho_{TT}\left(m\right) \end{array}$$

In high-SNR regions, demapping errors due to noise effects become negligible, which improves the accuracy of channel estimate $H_{CSI}\left(m,k\right)$. Thus, the correlation coefficient between $H_{CSI}\left(m,k\right)$ and $\hat{H}\left(m,k\right)$ is close to 1, which is the maximum correlation coefficient. Likewise, the channel estimate $H_{TT}\left(m,k\right)$ generally has a high correlation coefficient with the initial channel estimate $\hat{H}\left(m,k\right)$ but has a lower correlation coefficient than $H_{CSI}\left(m,k\right)$ owing to the removal of the channel information of $\hat{H}\left(m,k\right)$ after the *L*-th tap.

The TRFI scheme is affected by the accuracy of the initial channel estimate. The initial channel estimate is reduced in accuracy by demapping errors at low SNRs; conversely, it is improved in accuracy by the reduction of demapping errors at high SNRs. Therefore, $H_{CSI}\left(m,k\right)$ obtained through the CSI can be used as an indicator for selecting the TRFI.

**Figure 3:**
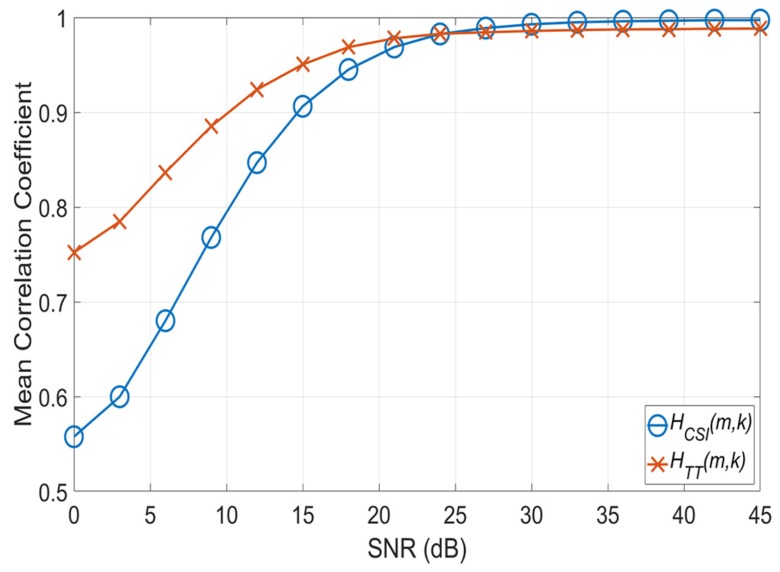
Mean correlation coefficient according to SNR.

Figure 3 shows the mean correlation coefficients $E\;\left[\;\sum_{l=1}^{m}\;\rho_{j}\;\left(l\right)\;\right]$ between $\hat{H}\left(m,k\right)$ and $H_{j}\left(m,k\right)$ according to the SNR, and it proves Equations (14) and (15). As shown in Figure 3, before 24 dB, we can observe that $H_{TT}\left(m,k\right)$ has a higher mean correlation coefficient than $H_{CSI}\left(m,k\right)$. After 24 dB, both channel estimates have high mean correlation coefficients. However, $H_{CSI}\left(m,k\right)$ approaches 1, whereas $H_{TT}\left(m,k\right)$ maintains a value less than 1. Therefore, $H_{CSI}\left(m,k\right)$ has a higher correlation coefficient than $H_{TT}\left(m,k\right)$.

## 5. Complexity Analysis

In this section, a complexity analysis of the proposed SLS scheme is performed for comparison with other channel estimation schemes such as TRFI and DD-TT. We employed an analytical method using the standard Landau notation, which is widely used to calculate the efficiency of an algorithm [19]. The Landau notation provides a high level of complexity without having to drill down into the detail of counting floating-point operations per second or individual operations.

Because the SLS scheme is sequentially conducted on a symbol-by-symbol basis in the packet, the complexity analysis on one OFDM symbol can be extended linearly to calculate the complexity of the entire packet. Assuming that the total number of subcarriers to be estimated in one OFDM is *N*, the complexity of each step of the SLS scheme can be simplified as shown in Table 3. Table 3 shows the complexity for the corresponding steps in the flowchart of Figure 2.

The SLS scheme basically consists of Steps ①∼④ to select a better scheme. The SLS scheme performs 1D cubic spline interpolation on the number of indexes *R* belonging to $K_{S}$ to obtain $H_{CSI}\left(m,k\right)$. The 1D cubic spline interpolation with *R* points has the complexity O(R+logR) [29]. In addition, time truncation using an FFT matrix is performed to obtain the channel estimate $H_{TT}\left(m,k\right)$ of the DD-TT. The FFT matrix size is scaled as N×L, where *L* represents the channel order. The channel estimate $H_{TT}\left(m,k\right)$ is obtained by the multiplication of the FFT matrix and the initial channel estimate vector; thus, the step of performing DD-TT has a complexity of O(2·L·N). If TRFI is selected, then Steps ⑥ and ⑦ are performed additionally. In ⑦, *P* is the number of initial channel estimates that have not passed the time reliability test, and it is determined by a number from 0 to 48 depending on the test result.

Overall, the SLS scheme has a linear time complexity O(N) in one OFDM symbol. In the IEEE 802.11p standard, because *N* is a fixed value, the complexity of the SLS scheme depends on the *M* OFDM symbols that comprise one packet, i.e., the SLS scheme has the time complexity O(M).

TRFI and DD-TT perform Steps ①, ⑥, and ⑦, and Steps ① and ③, respectively, as shown in Table 4. Based on the analysis of the step-by-step complexity in Table 3, TRFI and DD-TT also have a linear time complexity O(N) in one OFDM symbol, and are affected by the number of OFDM symbols in the packet.

Consequently, the SLS scheme has a linear time complexity like the DD-TT and TRFI schemes. However, in more detail, as SLS performs additional steps compared with TRFI and DD-TT, the computational speed is TRFI < DD-TT < SLS.

## 6. Simulation Results and Discussion

In this section, we compare the performance of the SLS scheme with that of other channel estimation techniques: the STA, TRFI, DD-TT, and PLS schemes. The PLS scheme is configured to select DD-TT and TRFI on a packet-by-packet basis. The performances were analyzed using a link-level simulator that complies with the IEEE 802.11p standard. The simulation channel environment is assumed to be V2V Expressway Oncoming and V2I Urban Canyon, and the channel model used for the simulation is shown in Table 2.

**Figure 4:**
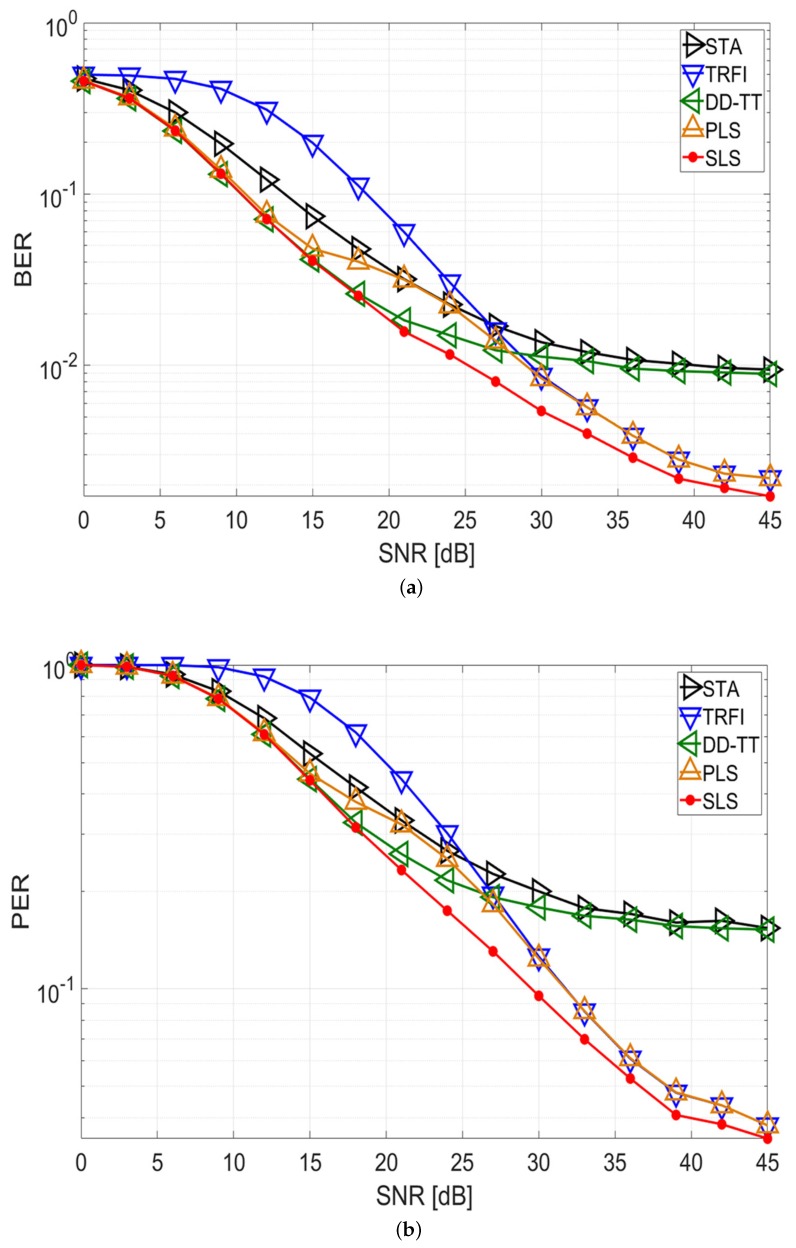
Performance analysis of STA, TRFI, DD-TT, PLS, and proposed SLS schemes (V2V channel, QPSK 1/2, 50 symbols). (**a**) BER performance; (**b**) PER performance.

**Figure 5:**
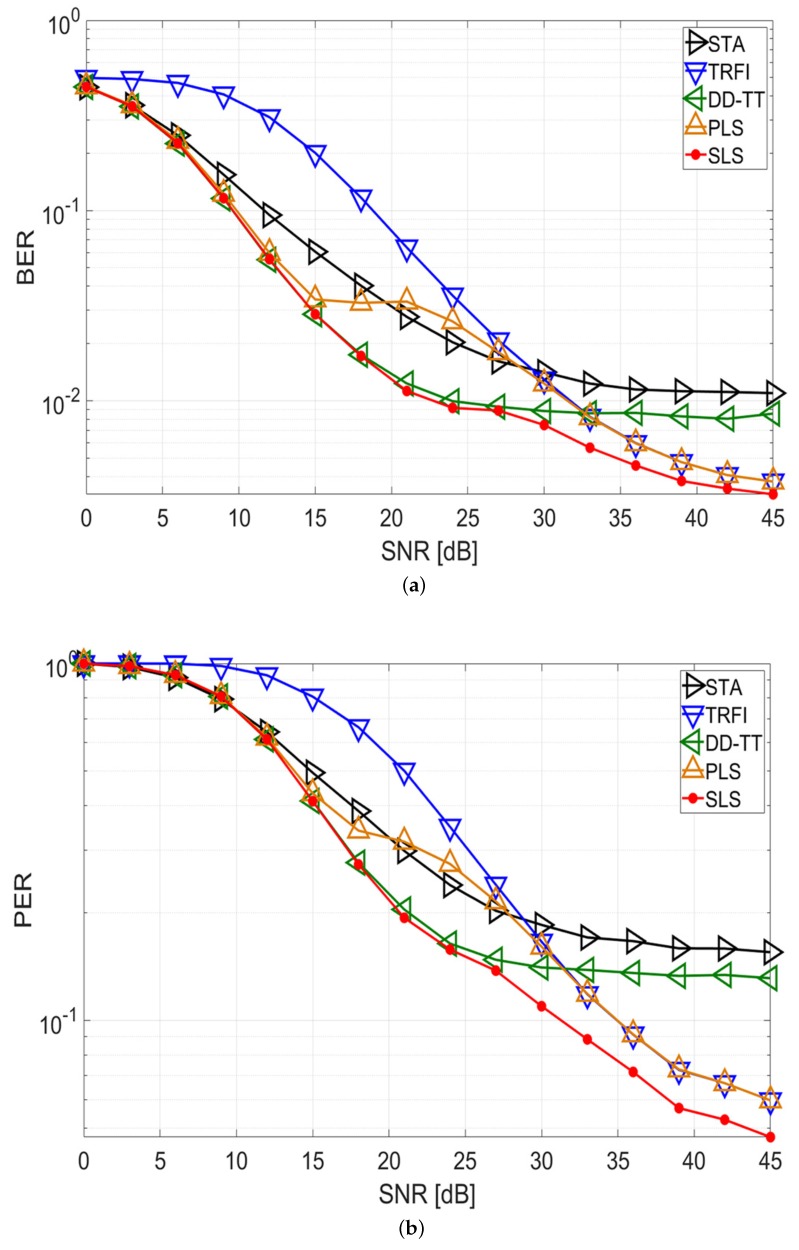
Performance analysis of STA, TRFI, DD-TT, PLS, and proposed SLS schemes (V2I channel, QPSK 1/2, 50 symbols). (**a**) BER performance; (**b**) PER performance.

Figure 4 and Figure 5 show the BER and PER performance of quadrature phase shift keying (QPSK) with code rate 1/2 in the V2V and V2I channel environments, respectively. It is assumed that a packet consists of 50 symbols.

For the V2V simulation in Figure 4, STA and DD-TT show that an error floor occurs in high-SNR regions, and TRFI achieves a lower error performance than the other schemes in low-SNR regions for both the BER and PER performances. The PLS scheme has satisfactory performance as a whole, but the performance degradation occurs between 18 and 24 dB compared with DD-TT. This means that the selected channel estimation technique at the beginning of the packet cannot accurately estimate the channel variation to the end of the packet. Meanwhile, the proposed SLS scheme follows the performance of DD-TT up to 18 dB, can achieve a performance gain of about 5–7 dB at a BER of 2 × $10^{-2}$, and offers a gain of about 3–6 dB at a PER of 3 × $10^{-1}$ compared with STA, TRFI, and PLS. After 18 dB, the SLS scheme performs better than the other schemes and offers a performance gain of about 2–3 dB at a BER of 5 × $10^{-3}$ and a PER of 9 × $10^{-2}$ compared with TRFI and PLS. These results demonstrate that the proposed SLS scheme selectively uses DD-TT and TRFI on a symbol-by-symbol basis depending on the channel condition, thereby improving performance.

For the V2I simulation in Figure 5, the BER and PER performances of the proposed scheme are similar to those in the V2V simulation. For both BER and PER, STA and DD-TT exhibit good performance at low SNRs, while TRFI performs very well at high SNRs. PLS performs worse than DD-TT between 18 and 30 dB, and the SLS scheme shows the performance of DD-TT up to 18 dB. After 18 dB, the SLS scheme provides the better performance compared with the other schemes, and can achieve a performance advantage of about 4 dB at a BER of 5 × $10^{-3}$ and a PER of 8 × $10^{-2}$ over the TRFI and PLS schemes. This indicates that the SLS scheme shows good performance in various channel environments.

**Figure 6:**
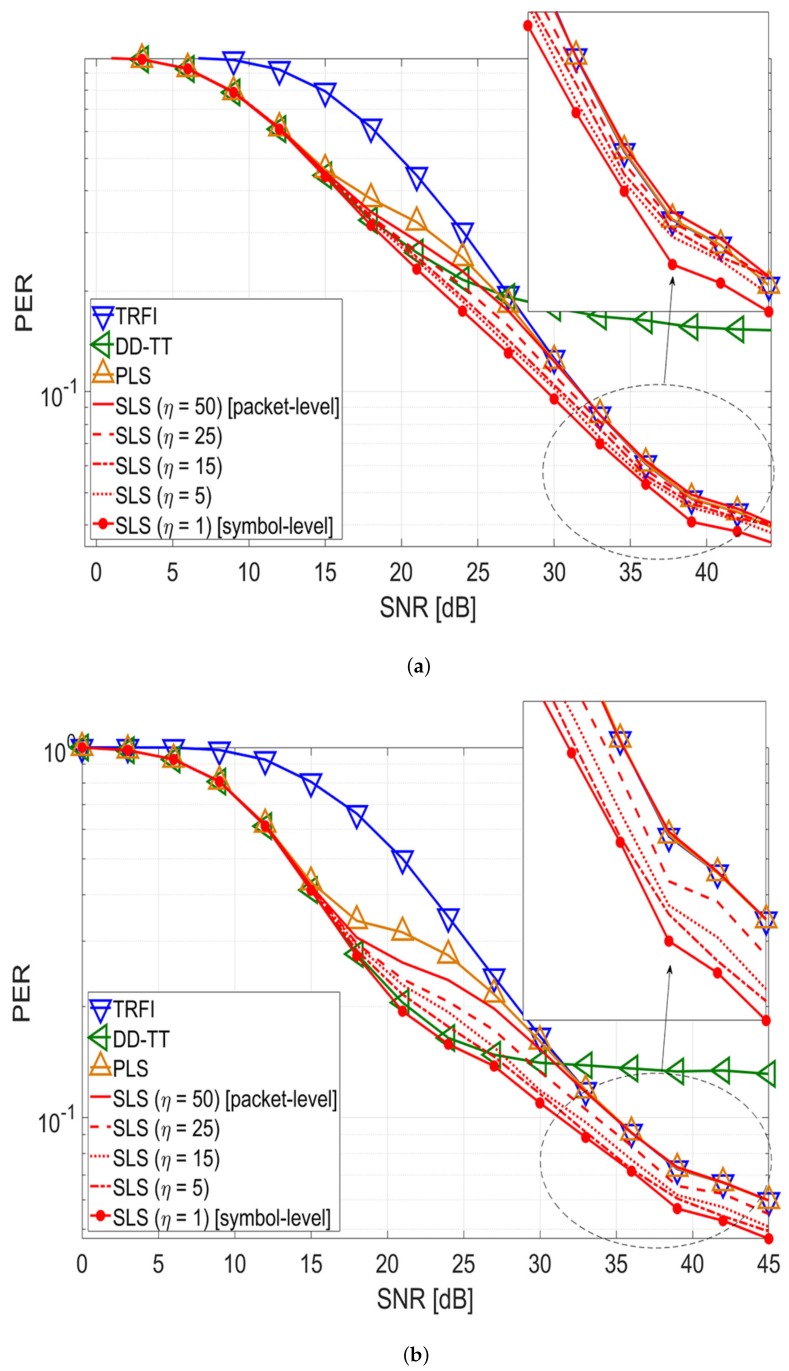
PER performance analysis according to the selection period of the SLS scheme (V2V/V2I channel, QPSK 1/2, 50 symbols). (**a**) V2V channel environment; (**b**) V2I channel environment.

**Figure 7:**
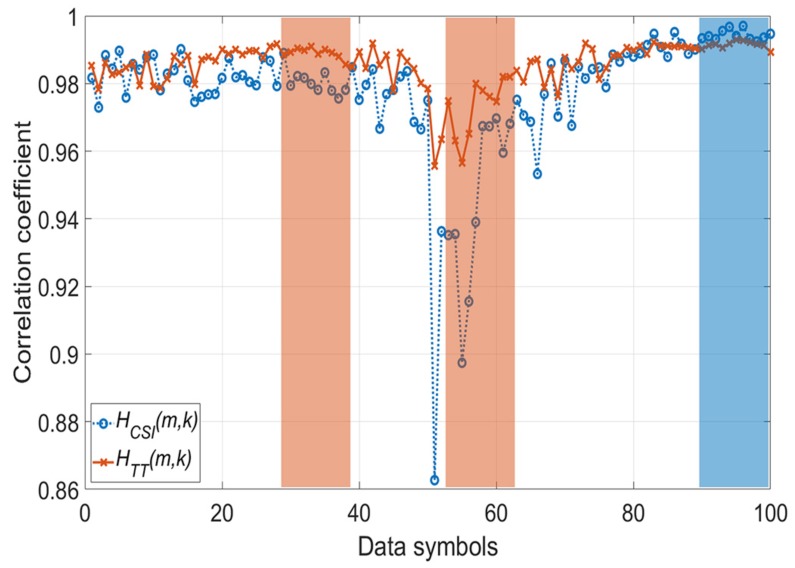
Correlation coefficient (SNR: 24 dB).

Figure 6 shows the PER performance according to the selection period in the SLS scheme. When the coherence time is defined as a bandwidth with a correlation of 0.9, the V2V and V2I channels according to the scenario in Table 2 have a coherence time of about 118.72 and 102.47 μ*s*, respectively [30]. Because the symbol duration of one OFDM is 8 μ*s* in IEEE 802.11p, the selected channel estimation scheme in the *m*-th OFDM symbol is valid up to 12∼14 symbol intervals. Figure 7 shows the correlation coefficients between the initial channel estimate $\hat{H}\left(m,k\right)$ and two channel estimates for each OFDM data symbol at an SNR of 24 dB. Assuming a packet consists of 50 symbols, Figure 7 shows the duration of two packets. In Figure 3, the mean correlation coefficients of $H_{CSI}\left(m,k\right)$ and $H_{TT}\left(m,k\right)$ intersect at 24 dB; thus, the selective use of TRFI and DD-TT occurs most frequently at around 24 dB. As shown in Figure 7, the correlation coefficients of DD-TT in the red boxes are consistently higher than that of CSI, and the blue box is the reverse. Therefore, one channel estimation scheme is chosen from the beginning to the end of the box, and in this interval, the results are the same whether the SLS scheme is performed once or on a symbol-by-symbol basis.

Based on this analysis, simulations are performed by changing the selection period of the SLS scheme within the packet. The selection period is defined as $\eta \times T_{OFDM}$, where η≜{1,5,15,25,50}. As shown in Figure 6, in both the V2V and V2I channel environments, SLS with η = 1 (symbol-level selection) provides the best performance, and the performance decreases with increasing η. When η = 5 and η = 15, SLS shows a performance similar to that of symbol-level selection. Interestingly, SLS with η = 25 and η = 50 (packet-level selection) provides a higher performance than the PLS scheme. Even with the same selection period with PLS, the SLS scheme offers better performance than the PLS scheme. This means that the criterion for selecting a better technique of SLS is superior to that of PLS.

Consequently, as η grows, the complexity of the SLS scheme is reduced, and the performance decreases. However, by choosing the appropriate η depending on the channel environment, the SLS scheme can reduce the complexity while minimizing the performance degradation compared with symbol-level selection.

Finally, link budget analysis is performed to confirm the effective communication range of the proposed SLS scheme in a practical vehicular network. By the simulation results of Figure 4 and Figure 5, the SNR region where the selective use of DD-TT and TRFI occurs frequently in the proposed scheme is 18–45 dB in the V2V channel and 24–45 dB in the V2I channel. Therefore, the most performance gain occurs in this SNR region, and the link budget analysis is performed for this portion. The link budget expression for SNR is as follows:

(16) $$SNR=P_{r}\left(d\right)-N,$$

where $P_{r}\left(d\right)$ represents the received power according to distance *d*, including transmission power, antenna gain, and pass loss. *N* is the thermal noise including the noise figure. The inverse calculation of Equation (16) yields an approximate communication range. We assume the transmit power, antenna gain, and the noise figure are set to 23 dBm, 3 dB, and 9 dB, respectively, and the WINNER II path-loss model [31] is applied to consider the practical network situation.

Applying the set parameters to Equation (16) to obtain the pass loss value and using this value to obtain the distance from the WINNER II pass-loss model, the effective communication range of the V2V channel is 35–135 m and the V2I channel is 45–135 m. Therefore, the proposed SLS scheme within this range (35–135 m) can get the highest performance gain, and better performance can be obtained by using the SLS scheme than by using the existing channel estimation method alone.

There are three discussions of the simulation results. The first discussion is related to the performance of the proposed SLS scheme. The proposed SLS scheme in Figure 4 and Figure 5 provide superior performance over the entire SNR region compared with STA, TRFI, DD-TT, and PLS on both V2V and V2I channels in terms of BER and PER. The performance gain of the proposed scheme is higher than that of the conventional schemes by detecting the channel variations within the packet and appropriately selecting the better technique on a symbol-by-symbol basis between DD-TT and TRFI.

The second discussion is related to the trade-off between performance and complexity according to the algorithm’s selection period within a packet. If the computational complexity is large, the implementation feasibility in hardware is very poor. In Table 3 and Table 4, the proposed SLS scheme has a linear time complexity and the performance reduction is not significant even if the selection period of the proposed scheme is increased as shown in Figure 6. Therefore, it is feasible to switch the two techniques in terms of hardware implementation, and the proposed scheme can cope with rapid channel variations.

The third discussion is related to the effective communication range of the proposed SLS scheme in the live network. Performance analysis in the live network over a link budget is important in determining whether the proposed SLS scheme satisfies the required performance on a given communication link. The link budget analysis shows that the proposed SLS scheme provides the best performance in the range between 35 and 135 m compared with when using the other schemes alone.

## 7. Conclusions

In this paper, we proposed a novel channel estimation scheme that selectively uses DD-TT and TRFI to overcome time-varying characteristics in vehicular environments. To adaptively select TRFI and DD-TT, the proposed SLS scheme was based on the correlation coefficient between channel estimates and uses the better channel estimation scheme on a symbol-by-symbol basis instead of the entire packet.

The SLS scheme was compared with previous channel estimation schemes through simulations. The simulation results demonstrated that the SLS scheme can be selectively used between TRFI and DD-TT on a symbol-by-symbol basis in the packet, thereby improving error performance. In QPSK modulation under V2I and V2V channel environments, the SLS scheme can achieve a performance gain of about 2–4 dB at PER compared with other schemes.

In terms of the computational complexity, the SLS scheme has a linear time complexity with respect to the number of OFDM symbols constituting a packet based on a complexity analysis. In addition, we analyzed the trade-off between the performance and complexity according to the selection period of the SLS scheme. As the selection period increases, the complexity and performance of the SLS scheme decreases. However, a proper selection period depending on the channel environment can reduce the complexity and minimize performance degradation over symbol-level selection.

We also analyzed the effective communication range of the proposed scheme through the link budget analysis in the live network. In the link budget analysis, it was confirmed that the distance of the SNR region where DD-TT and TRFI are frequently switched is 35–135 m for the V2V channel and 45–135 m for the V2I channel. Within this range, the proposed scheme achieves maximum gain, and using the proposed technique yields better performance than using other techniques alone.

## Figures and Tables

**Table 1:** OFDM system parameters in IEEE 802.11p.

Parameter	Value
Carrier frequency: $f_{c}$	5.9 GHz
Bandwidth: *B*	10 MHz
The number of data subcarriers: $N_{d}$	48
The number of pilot subcarriers: $N_{p}$	4
The number of total subcarriers: $N_{tot}$	64
The length of CP: *G*	16
Sampling time: $T_{s}$	0.1 μs
Symbol duration: $T_{OFDM}$	8.0 μs

**Table 2:** Parameters for the channel model.

Scenario	Distance between TX & RX (m)	Velocity (km/h)	Doppler Shift (Hz)	Maximum Excess Delay (μ*s*)
V2V Expressway Oncoming	300–400	104	1000–1200	0.3
V2I Urban Canyon	100	32–48	300	0.5

**Table 3:** Time complexity of steps for the SLS scheme.

Steps	Time Complexity
① Initial Channel Estimation	O(2·N)
② 1D Cubic Spline Interpolation	O(R+logR)
③ Time Truncation	O(2·L·N)
④ Correlation Coefficient	2·O(N)
⑥ Time Reliability Test	O(2·N)
⑦ 1D Cubic Spline Interpolation	O(P+logP)
Overall Steps	O(N)

**Table 4:** Comparison of time complexity.

Schemes	Composition	Total Complexity
TRFI	①, ⑥, ⑦	O(N)
DD-TT	①, ③	O(N)
Proposed SLS	①∼④	O(N)
